# Avian Influenza Virus Tropism in Humans

**DOI:** 10.3390/v15040833

**Published:** 2023-03-24

**Authors:** Umarqayum AbuBakar, Lina Amrani, Farah Ayuni Kamarulzaman, Saiful Anuar Karsani, Pouya Hassandarvish, Jasmine Elanie Khairat

**Affiliations:** 1 Institute of Biological Sciences (ISB), Faculty of Science, Universiti Malaya, Kuala Lumpur 50603, Malaysia; 2 Tropical Infectious Diseases Research and Education Center, Universiti Malaya, Kuala Lumpur 50603, Malaysia

**Keywords:** influenza A virus, avian influenza virus, virus tropism in humans, antiviral, vaccine

## Abstract

An influenza pandemic happens when a novel influenza A virus is able to infect and transmit efficiently to a new, distinct host species. Although the exact timing of pandemics is uncertain, it is known that both viral and host factors play a role in their emergence. Species-specific interactions between the virus and the host cell determine the virus tropism, including binding and entering cells, replicating the viral RNA genome within the host cell nucleus, assembling, maturing and releasing the virus to neighboring cells, tissues or organs before transmitting it between individuals. The influenza A virus has a vast and antigenically varied reservoir. In wild aquatic birds, the infection is typically asymptomatic. Avian influenza virus (AIV) can cross into new species, and occasionally it can acquire the ability to transmit from human to human. A pandemic might occur if a new influenza virus acquires enough adaptive mutations to maintain transmission between people. This review highlights the key determinants AIV must achieve to initiate a human pandemic and describes how AIV mutates to establish tropism and stable human adaptation. Understanding the tropism of AIV may be crucial in preventing virus transmission in humans and may help the design of vaccines, antivirals and therapeutic agents against the virus.

## 1. Introduction

Human influenza is a contagious acute respiratory illness caused by the influenza virus via direct infection, mainly in the respiratory tract, that usually exhibits symptoms including fever, headache, chills, myalgia, dry cough and sore throat [1,2]. Influenza viruses are a group of segmented, negative-sense RNA viruses that belong to the *Orthomyxoviridae* family that are further classified into Alphainfluenzavirus (species influenza A virus), Betainfluenzavirus (species influenza B virus), Deltainfluenzavirus (species influenza D virus) and Gammainfluenzavirus (species influenza C virus) [3]. Influenza A viruses have the ability to infect various host species, while influenza B and C mainly infect humans, and the new type of influenza D is known to circulate in cattle [4]. Influenza A and B can cause a predictable seasonal flu epidemic in humans every year. However, the influenza A virus (IAV) from zoonotic reservoirs can sometimes cause a deadly pandemic [5].

The natural reservoir of IAV is birds, where the virus can adapt and transmit to poultry and mammalian hosts including humans and pigs [6]. The transmission of IAV from birds to humans has been associated with high pathogenicity of the novel virus that causes high severity and mortality. The deadliest pandemic in recent history, the influenza pandemic 1918 (H1N1) or Spanish Flu, which was most likely caused by an avian influenza virus (AIV), killed between 50 and 100 million people worldwide [7]. Additionally, a subtype of AIV, H5N1, was also reported to infect humans and caused a high mortality rate, estimated at more than 50% of infected patients [8]. Avian influenza, commonly referred to as fowl plague, is an infectious disease caused by AIV in poultry. Human avian influenza is an acute respiratory tract infection caused by certain strains of subtypes of AIV. The first severe fowl plague in chickens was first reported in Italy in 1878 [9], and it was later confirmed to be caused by a virus identified as the influenza A virus in 1955 [10]. In 1981, such a disease was officially named avian influenza at the first international avian influenza conference [11]. AIV subtypes that have caused human avian influenza in the past and present include H1, H2, H3, H5, H6, H7, H9 and H10 [12].

AIV enters the respiratory tract through the nose, mouth or even eyes in humans and continuously travels along the respiratory tract to search for suitable host cells [4]. Types of human cells infected by AIV are respiratory epithelial cells [13,14], intestinal epithelial cells [15,16], immune cells [17,18] and nerve cells [19]. AIV does not typically transmit directly to humans and is assumed to require consecutive adaptation before transmitting efficiently among humans [20]. AIV tropism in humans depends on various host and viral factors for efficient replication, which means that without proper interaction with the human host cell, AIV cannot replicate. The AIV tropism determinants can be found at each replication step, from viral entry into cells to virus progeny release from infected cells [21]. This review discusses the key determinants of AIV tropism in humans by both virus and host factors. Understanding AIV’s tropism may be essential for preventing and controlling virus transmission across humans as well as enhancing the development of antivirals and therapeutics against the virus.

## 2. Influenza A Virus Structure

IAV virions are enveloped, pleiomorphic particles in the shape of spheres with a diameter of 100 nm, or filaments with a diameter from 100 nm to 30 µm [22]. They are composed of three main subviral components: an envelope, a layer of matrix 1 (M1) proteins and a viral ribonucleoprotein (vRNP) core. Their outer layer (envelope) consists of a lipid bilayer membrane derived from the host cell membrane and acquired during the budding process; it contains two integral transmembrane glycoproteins forming spikes on the surface of the virus (hemagglutinin (HA) and neuraminidase (NA)) and a transmembrane ion channel matrix 2 (M2) protein. Experts in the classification and taxonomy of viruses divide IAV into subtypes based on their surface glycoproteins HA and NA. To date, 18 different HA (H1–H18) and 11 NA (N1–N11) proteins have been identified, resulting in 198 potential IAV subtype combinations [23]. However, it is unknown whether H17 and H18, as well as N10 and N11, which belong to influenza virus-like bat viruses, can reassort with other avian or human IAVs. Underlying the viral lipid membrane is a protein layer composed of the inner surface envelope M1 protein, which forms the shell and serves as a structural support for viral particles and the nuclear export protein (NEP/NS2), which contributes to the nuclear export of vRNP [24].

**Figure 1:**
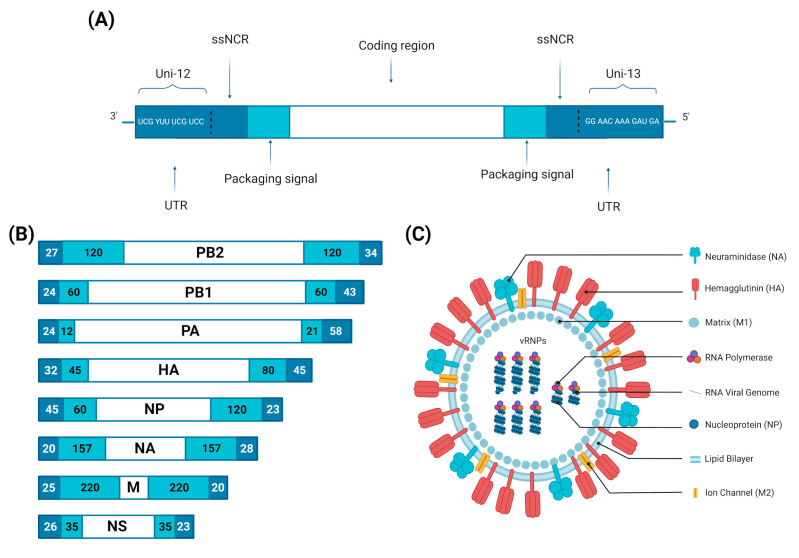
Influenza A virus virion structure and genome organization. (**A**) Schematic organization of a gene segment (vRNA). The IAV genes consist of three parts: 1. Untranslated regions (UTRs) comprise the universal primers uni-12 (12nt) and uni-13 (13nt) at the end of 3′ and 5′ of each segment, which are highly conserved among the eight gene segments and among all IAV viral strains; and the segment-specific non-coding regions (ssNCRs); 2. Packaging signals; and 3. Coding region. (**B**) IAV Genome organization: The eight negative-sense, single-stranded RNA segments of IAV genome code for PB2, PB1, PA, HA, NP, NA, M and NS, respectively (indicated in white boxes). The darker blue colour boxes at the end of each vRNA segment indicate the 3′ and 5′ untranslated regions (UTRs); the lighter blue colour boxes indicate the packaging signals. The numbers inside the blue boxes represent the nucleotide length for the UTRs and the packaging signals. (**C**) IAV virion structure: IAV comprises three components: an envelope made of a lipid bilayer containing three transmembrane proteins (HA, NA and M2 ion channel); a layer of the inner surface envelope M1; and a core made of eight vRNA segments, packaged together to form vRNP. (Created with BioRender.com).

The IAV genome is made of eight segments of negative-sense, single-stranded viral RNA (vRNA), which are varied in size and encode for IAV’s main proteins: polymerase basic 2 (PB2), polymerase basic 1 (PB1), polymerase acidic (PA), HA, nucleoprotein (NP), NA, M (M1 and M2) and non-structural protein (NS) (NS1 and NEP/NS2), as indicated in Figure 1 and Table 1 [25]. Each segment is packed with NP and linked with RNA-dependent RNA polymerase (RdRp), and the complex of PA, PB1 and PB2 to form a separate vRNP complex [26,27]. The viral gene segments organize themselves in a similar fashion for all orthomyxoviruses. Each vRNA segment has a coding region, flanked on both sides by untranslated regions (UTRs) which range from 19 to 58 nucleotides in length [28]. These contain regions specific to each gene segment, segment-specific non-coding regions (ssNCRs), as well as a conserved sequence common to all vRNAs: the 12 nucleotides of the 3′ end and the 13 nucleotides of the 5′ end are conserved for all the gene segments of all the influenza A strains. Partially complementary, they combine to form a secondary structure, a hairpin or corkscrew, found at the vRNP level [29,30].

## 3. Zoonotic Transmission of Influenza A Viruses

**Figure 2:**
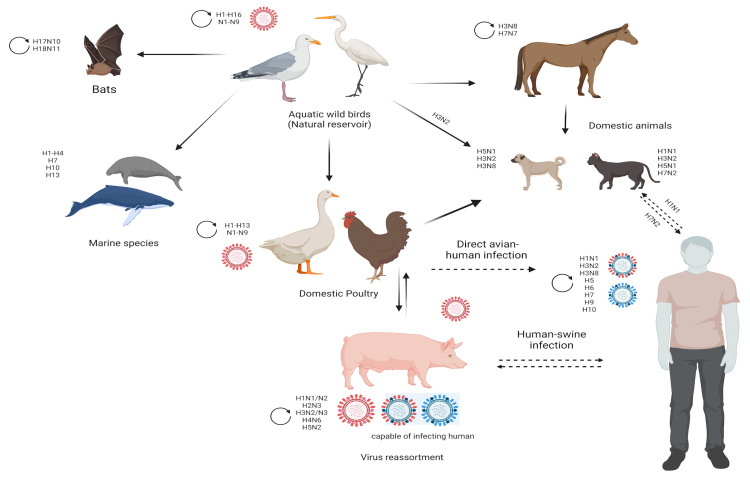
Schematic diagram showing the host range of influenza A viruses. Reservoirs and interspecies transmission events of influenza A viruses and the subtypes involved in these events. Aquatic wild birds represent the natural reservoir of influenza A viruses, from which they can be transmitted to a variety of other hosts. Circled arrows represent continuous virus circulation among wild birds, domestic birds, domestic animals, bats, horses, pigs and humans. (Created with BioRender.com).

In general, IAV evolution, the vast biodiversity of host species and the host adaptation mechanisms (tissue tropism, host immune response, host receptor adaptation) are caused by the antigenic drift and antigenic shift of the virus. Antigenic drift refers to the gradual accumulation of mutations in the genes that code for the surface proteins of the virus, HA and NA, which can alter the antigenic properties of the virus over time. These mutations can result in new strains of the virus that are able to evade the host’s immune system and cause seasonal influenza outbreaks. Antigenic shift, on the other hand, is a more dramatic process that occurs when two or more different influenza viruses infect the same host and reassort genetic material, resulting in the emergence of a novel strain of the virus that has a completely new combination of surface proteins. This process can lead to pandemics of influenza, as the human population has little or no immunity to the new strain [44]. Both antigenic drift and antigenic shift contribute to a wide variety of avian and mammalian animal species infections, which increases the probability of cross-species transmission events, from which the IAV can spread to humans [31,45]. As illustrated in Figure 2, IAV has the ability to infect swine, avian, feline, equine and canine species, as well as humans and other mammalian species [46].

### 3.1. Swine

Pigs are excellent mixing vessels for IAV of different host origins as they are highly susceptible to co-infections with both avian and human influenza viruses due to the mixed sialic acid receptor (α-2,3 and α-2,6) distribution on the glycocalyx of epithelial cells lining the porcine trachea [45,47]. It is unknown if swine IAV infections existed prior to 1918, when swine IAV was clinically identified in the context of the 1918 pandemic [48]. Since then, swine IAV has been consistently recognized as a serious disease with significant economic and public health implications [47]. Swine IAV H1N1 was initially isolated by Shope and Lewis in 1930 [12]. Since then, IAVs of various subtypes (H1N1, H1N2 and H3N2) have been isolated from pigs around the world, with some subtypes causing enzootic infections and others causing only localized outbreaks with no sustained spread [49].

### 3.2. Equine

Equine influenza is a respiratory viral disease which can affect all *Equidae* (horses, ponies, donkeys, etc.), as well as animals resulting from their crossbreeding (mules) [50,51]. It is highly contagious and spreads rapidly; an unvaccinated population has an almost 100% infection rate [52]. Mortality is low, except in young or weakened equids [51]. It is hypothesized that avian influenza strains were the progenitors of equine influenza viruses (EIVs) [53]. Two IAV subtypes are known to infect horses: H7N7 and H3N8. It appears that the H7N7 subtype is no longer in circulation, since the last epidemic occurred in the late 1970s. Since then, the H3N8 subtype has been the most isolated from sick horses [54].

### 3.3. Canine and Feline

Canine and feline influenzas are contagious acute respiratory illnesses in dogs and cats caused by a variety of influenza viruses including equine influenza virus H3N8, swine influenza virus H3N2 and H1N1, as well as the low-pathogenic avian influenza virus (LPAIV) H7N2 and the highly pathogenic avian influenza virus (HPAIV) H5N1 [55,56]. In addition to these subtypes, cats and dogs can also be infected with other IAV subtypes, primarily of avian origin or resulting from genetic reassortments (i.e., H5N6, H5N2, H3N1), as a result of co-infections with various avian, swine and human IAVs [55]. In 2011, the transmission of the 2009 H1N1 IAV from humans to cats was reported, causing severe pneumonia in cats [57]. In 2016, the New York City Department of Health and Mental Hygiene (NYC DOHMH) documented the first cat-to-human transmission of IAV H7N2 [58].

## 4. Avian Influenza Virus

Avian influenza viruses (AIVs) can adapt to many host habitats thanks to the vast diversification of birds (more than 10,000 species) [59]. All AIVs are thought to have originated from migratory birds near wetlands or other aquatic habitats, particularly those belonging to the orders *Anseriformes* (waterfowl) and *Charadriiformes* (shorebirds) [60]. Along their migration route, they spread and interchange various viral strains, which causes antigenic drift and genome reassortment, and can result in the emergence of novel influenza strains [8]. AIVs are classified into 16 HA and 9 NA subtypes based on the antigenic variation of their surface glycoproteins, which can yield up to 144 possible HA/NA combinations identified in birds [61,62]. Nearly all AIV subtypes are present in wild aquatic birds and can spread sporadically to domestic poultry and/or mammals through their saliva, faeces and nasal secretions [63]. In poultry, the pathogenicity of AIV is classified into two pathotypes: low-pathogenic avian influenza (LPAI) and highly pathogenic avian influenza (HPAI) [64]. Only six subtypes of avian influenza viruses have been reported to cause infections in humans: H3 (H3N8), H5 (HPAI H5N1, H5N6 and H5N8), H6, H7, H9 (LPAI H9N2) and H10 viruses (Table 2) [65,66]. It is hypothesized that AIV spreads from poultry to humans primarily through direct contact with sick poultry or surfaces and objects contaminated by their faeces or secretions. Another speculation holds that AIV infects pigs first, and then the infection spreads to humans via contact with the infected pigs’ secretions, blood, skin and fur [67].

## 5. Key Determinants of AIV Tropism in Humans: Virus and Host Factors

**Figure 3:**
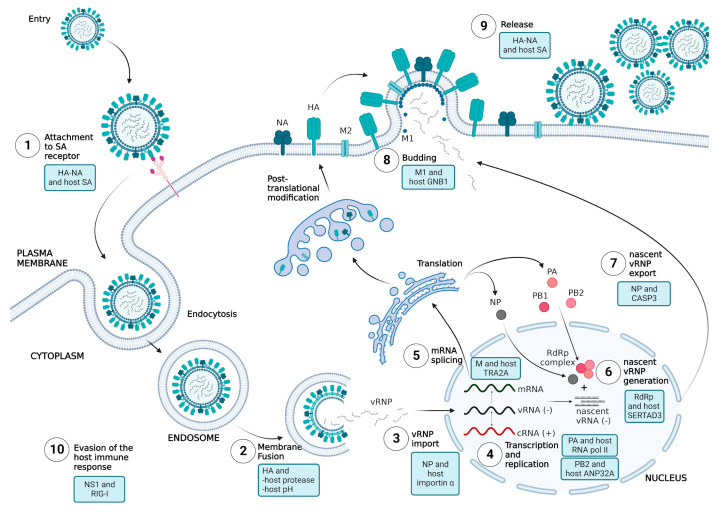
IAV life cycle and the key determinants of AIV tropism in the human host. (1) Upon viral entry in the respiratory tract, AIV attaches to the host sialic acid (SA) receptor by interacting with HA–NA, thus, inducing cell internalization via endocytosis. (2) The acidification of the late endosome (host pH) triggers a conformational change in cleaved HA (where HA is cleaved by host protease), which later causes membrane fusion and vRNP release into the cytoplasm. (3) Following that, vRNPs are transported into the nucleus via the host importin α/β-mediated nuclear import pathway. (4) In the nucleus, replication and transcription of viral RNA are proceeded by viral polymerases with the help of host RNA polymerase II and ANP32A. (5) After transcription, the viral mRNAs leave the nucleus to be transported to the host ribosome for translation. However, the viral pre-mRNA of M and NS undergo a maturation process in which the pre-mRNAs are spliced before the translation process. (6) In the nucleus, upon viral protein translation, NP binds to nascent viral RNA to prevent RNA degradation. PA, PB1 and PB2 are assembled as RdRp complex before binding with RNA-NP, thus, generating nascent vRNP. Then, nascent vRNPs are exported from nucleus to cytoplasm (7) and all nascent viral proteins are trafficked to the budding membrane. (8) The bud formation is initiated by the interaction of M1 with host machinery before (9) the nascent virus is released by the interaction of HA–NA with the host SA receptor. (10) Evasion of the host immune response. (Created with BioRender.com).

The interaction between the host and virus highlights some of the host factors that limit AIV replication at each stage in humans. However, mutations in an AIV can counteract these restrictions to enable efficient replication in human cells. This article discusses the key determinants of AIV’s ability to replicate efficiently in humans, based on both virus and host factors, in each stage of virus replication (Figure 3).

### 5.1. Attachment

The interaction of the viral transmembrane envelope proteins, hemagglutinin (HA) and neuraminidase (NA), with the host cell surface receptor, sialic acid (SA), during attachment and internalization is the critical determinant of AIV tropism in humans.

#### 5.1.1. HA and Host SA

HA is initially expressed as a precursor protein of HA0 before being cleaved by a host protease into a mature HA1–HA2 complex [91]. The mature HA1–HA2 complex consists of two domains, which are the globular head domain composed of HA1 (containing receptor binding site, RBS) and the stalk domain, primarily composed of HA2 (containing fusion peptide) [92]. The HA1 binds to cell-surface receptors and triggers viral internalization via endocytosis, whereas the HA2 facilitates virus–host cell membrane fusion via an alteration of the pH in the endosome [93]. The RBS in HA1 is relatively shallow and comprises at least four amino acids (Y98, W153, H183 and Y195), which are conserved in all AIV subtypes (H1–H16 subtypes). These amino acids are encircled by 130 loop, 150 loop, 190 helix and 220 loop structures [94,95]. Although all these loop and helix structures are present in all strains, their length and amino acid composition vary, and these differences are typically critical determinants in recognizing the type of receptor for binding [96].

The most established and well-known host cell surface receptor that binds to the RBS in HA1 is sialic acid (SA), specifically N-acetylneuraminic acid (Neu5Ac, also known as α2,6 SA) and N-glycolylneuraminic acid (Neu5Gc, also known as α2,3 SA), which is located on the terminal glycans of host transmembrane proteins [4]. Generally, AIV prefers to bind α2,3 SA, while human influenza viruses prefer to bind α2,6 SA [96]. Studies of SA distribution in humans revealed that α2,6 SA is more abundant in the upper respiratory tract (including nasopharynx and mucus-producing cells), whereas α2,3 and α2,6 SA are equally prevalent in the lower respiratory tract, which includes the trachea, lungs and bronchus [97]. Despite the presence of α2,3 Sas in the respiratory tract, AIV transmission to humans remains inefficient. This is possibly due to a lack of α2,3 SAs and effective host innate immune responses in the upper respiratory tract, which prevents AIV from traveling to the lower respiratory tract that contains more α2,3 SAs [97,98]. However, previous cases of AIV transmission in humans occurred as a result of direct contact or exposure to infected animals, causing a high viral load that could travel deeper into the lower respiratory tract while escaping the host antiviral response, allowing AIV to bind to an acceptable amount of α2,3 SAs and then lead to a viral pathological effect [46,99].

**Figure 4:**
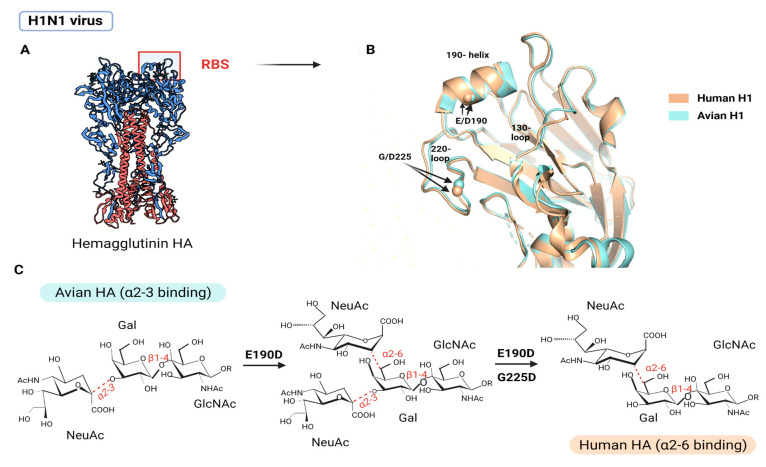
E190D and G225D substitution in H1N1. (**A**) Overall trimeric structure of HA of IAV H1N1. The location of RBS is marked. (**B**) Comparison of the structural characteristics and amino acid positions of avian (cyan) and human (beige) H1 RBS proteins. (**C**) The impact of specific receptor-binding variants on viral receptor specificity that facilitated the entry of H1N1 into the human cells. Adapted from ref. [101]. Created using Pymol (Schrodinger LLC) with PDB IDs 1RV0 and 1RVZ, and with BioRender.com.

**Figure 5:**
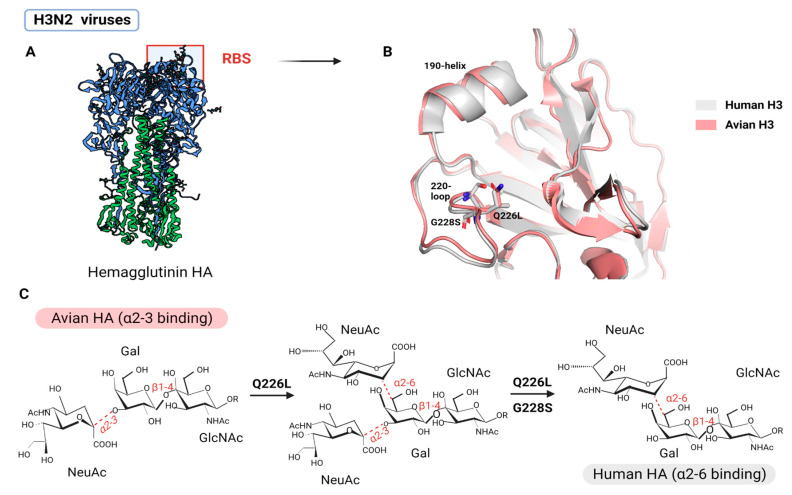
Q226L and G228S substitution in H3N2. (**A**) Overall trimeric structure of HA of IAV H3N2. The location of RBS is marked. (**B**) Comparison of the structural characteristics and amino acid positions of avian (pink) and human (white) H3 RBS proteins. (**C**) The impact of specific receptor-binding variants on viral receptor specificity that facilitated the entry of H3N2 into the human cells. Adapted from ref. [101]. Created using Pymol (Schrodinger LLC) with PDB IDs 6TZB and 1MQM, and with BioRender.com.

To achieve efficient and stable transmission between humans, AIV needs to switch binding preference from α2,3 SA to α2,6 SA via amino acid substitution at the RBS of HA1 [100]. Initially, the RBS must have distinct amino acids to accommodate the different conformations of α2,3 SA and α2,6 SA. In past influenza pandemics, at least two adaptive substitutions in RBS were required to shift the preference from α2,3 SA to α2,6 SA: E190D and G225D for H1N1 (1918 and 2009) and Q226L and G228S for H2N2 (1957) and H3N2 (1968) [96]. In H1N1, E190D and G225D substitutions influence receptor-binding specificity via two slightly distinct but correlated mechanisms: mutations within the 190 helix (located at the top of the RBS) improve stability via hydrogen bonding and remove side chains that potentially inhibit α2,6 SA binding, while mutations within the 220 loop (located toward the RBS base) influence the preferential adaptation from α2,3 SA to α2,6 SA [101] (Figure 4). In H2N2 and H3N2, the Q226L substitution established a hydrophobic environment incompatible with the α2,3 SA’s hydrophilic glycosidic oxygen but complementary to the α2,6 SA’s hydrophobic C6 atom, resulting in preferential binding to human receptors [102] (Figure 5). Furthermore, the G228S formed a hydrogen bond with SA, thus, enhancing HA’s affinity to α2,6 SA [103]. Despite being different variants, the broad effect of Q226L/G228S in H2 and H3 appears to be similar to G225D in H1, where adaptive variants act in close proximity to the SA glycosidic linkage to promote binding to α2,6 SA while reducing the preference for α2,3 SA through steric hindrance [101].

The possibility of a pandemic along with evidence of partial human adaptation has triggered the curiosity of researchers about the further adaptive mutations that may be required for AIVs to fully ‘jump’ the species barrier into humans, especially in the case of H5N1, H7N9, H6N1, H9N2 and H10N8. According to studies, H5N1 could establish persistent human-to-human and full airborne transmission via Q226L, N224 or G228S mutations [101]. However, current natural H5N1 and its variants have been reported to preserve dominant binding to α2,3 SA, showing that only partial adaptation to α2,6 SA has occurred. Furthermore, from 2016 to 2020, there was a significant decline in H5N1 human infections, lowering the probability of such evolution and the risk of a new pandemic [71]. Early H7N9 isolates were reported to possess a Q226L substitution linked to a pandemic-related receptor specificity switch mutation, but they retained significant binding to α2,3 SA [100]. According to de Vries et al., the variant requires simultaneous substitutions of three amino acids for a complete α2,6 SA switch specificity, involving either V186G/K–K193T–G228S or V186N–N224K–G228S [104]. Meanwhile, in the fifth wave of human infection by H7N9, Pu et al. discovered six more substitutions in the HA1 RBS that may contribute to the α2,6 SA switch specificity: A118T, S123N, A131V, R136K, L173I and M232I [105]. This wave was worse than the previous and following waves, spreading fastest from September 2016 to April 2017 with 623 confirmed cases [106]. Thus, current circulating H7N9 viruses have attracted a lot of attention due to the increased potential for a pandemic. In the cases of H6N1, H9N2 and H10N8, these viruses have a low risk of infecting humans and cause only mild symptoms. Nonetheless, their pandemic risks must not be overlooked. H6N1 and H10N8 could achieve complete human-type receptor specificity by only substituting G225D, Q226L or G228S [94,104]. Meanwhile, H9N2 has been reported to be able to reassort with other circulating AIVs including H5N1, H7N3 and H7N9 [101].

#### 5.1.2. NA and Host SA

Another AIV glycoprotein that interacts with SA, NA, has been chiefly focused on for its role in the exit of progeny virus from infected cells, where recent growing data support an essential role of NA during the virus attachment and internalization process [92]. The catalytic activity of NA removes sialylated ‘decoy’ receptors on mucin, cilia and glycocalyx, allowing the virus to travel smoothly across the cell surface and efficiently access functional receptors on the surface of the host cell [107]. In contrast with the catalytic activity of NA, it was proposed that NA also interacts with SA through direct receptor binding. The NA binding site is either at the same catalytic site or near it, and is referred to as the second binding site. Although the biological role of receptor binding via NA is still unclear, it is thought to be critical for viral entry.

The substitution of D151G, D151A, D151N or T148I near the NA active site in H3N2 [108,109] or G147R in N1 NA [110] correlates with the receptor binding acquisition. The fact that the entry and infection of AIV with NA D151G in MDCK cells can be blocked by NA inhibitors [111] supports the crucial role of NA active site-associated receptor binding in virus entry. Concerning the complementary and opposing effects of HA and NA on SA binding, the relative activity of the two proteins must be balanced, preventing one’s role from overshadowing the other’s and preserving the capacity to successfully infect and be released from cells [92]. The HA avidity for α2,6 SA must be high enough to allow binding before the NA may cleave the receptor in order to attach and enter the host cell. In contrast, HA binding to α2,6 SA cannot be too strong because the NA must be able to cleave the receptor to release new progeny virions and avoid aggregation at the cell surface [107]. Moreover, a functional balance between HA affinity and NA enzymatic activity with SA is necessary to facilitate airborne transmission between humans [4].

### 5.2. Membrane Fusion

Following viral internalization via endocytosis, the early endosomes containing virus particles interact with microtubules for retrograde traffic towards the microtubule’s organizing centers adjacent to the cellular nucleus via dynein motor proteins [112]. Upon reaching the nucleus, acidification of the late endosome due to the host intracellular degradation system [113] then activates the proton channel function of AIV matrix protein 2 (M2) to bring protons into the virion and triggers a conformational change in HA, allowing virus–host cell membrane fusion [114]. Based on previous studies focused on virus–cell membrane fusion, several key determinants of AIV tropism in humans are highlighted in this replication step: HA, host proteases and the pH of the host.

#### 5.2.1. HA and Host Proteases

At an early stage, viral HA is translated as a fusion-inactive precursor to avoid premature fusion and HA cleavage along the secretory pathway [115]. The proteolytic cleavage of the HA precursor (HA0) by the HA-processing proteases into HA1 and HA2 subunits generates the fusion peptide at the N terminus of HA2, therefore, gaining the ability to facilitate membrane fusion [116]. Hence, uncleaved HA is unable to result in membrane fusion. Since HA-processing proteases are not encoded in the virus genome, AIV utilizes proteases from the host cell for HA cleavage. The availability of appropriate host proteases is crucial for efficient virus replication and the severity of the infection [117]. Due to the specificity of the cleavage site, only proteases originating from specific tissues, organs or species can cleave and activate HA0, therefore, showing that the cleavage process is a key determinant of the tropism and pathogenicity of AIV in different types of tissues, organs and species. Based on previous studies, there are at least two types of human host proteases that can cleave HA0 from AIV: transmembrane serine proteases and secreted serine proteases [115].

The discovery of specific type II transmembrane serine proteases (TTSPs) capable of cleaving and activating HA from human-adapted subtypes has sparked a lot of interest in influenza studies. The transmembrane protease serine-2 (TMPRSS2) from the TTSP family and human airway trypsin-like protease (HAT) have been reported to accelerate trypsin-independent influenza virus propagation in vitro [115]. Both proteases are expressed in the human lung, suggesting that they might play an important role in influenza infection in vivo [118,119,120]. Other members of the TTSP family have also been discovered to cleave and activate the viral HA since the discovery of these two proteases. Chaipan et al. reported that TMPRSS4 could cleave and activate the HA from the 1918 pandemic influenza virus [121], while Okumura et al. discovered that mosaic serine protease large-form and a splice variant, TMPRSS13, can cleave and activate the HA from HPAI strains, giving an alternative to furin cleavage [122].

HA is also cleaved by a few secreted trypsin-like proteases. Secreted proteases that cleave HA from the H3 subtype (H3N2) include cellular trypsin, porcine mast cell tryptase, tryptase Clara and tryptase TC30 [3]. On the other hand, human mast cell tryptase was found to be incapable of activating HA [123]. Blood proteases, such as plasmin, urokinase, plasma kallikrein and thrombin, have also been found to cleave and activate HA [116]. The cleavage of HA from various subtypes and strains by these blood proteases indicated diversity in cleavage within a particular subtype. This is fascinating because all of the subtypes and strains studied have a monobasic cleavage site with little variation in the cleavage site region.

The involvement of co-infecting bacteria in HA activation has also been explored, as they may provide an additional supply of HA-cleaving proteases. Certain strains of *Staphylococcus aureus* were found to secrete proteases that may activate HA either directly or indirectly, and the inoculation of this protease with the virus improved pathogenicity in vivo [6,115,124]. It is exciting for future research to extend this investigation to determine if any other commonly found co-infecting bacteria give an additional supply of HA-cleaving proteases. Overall, it is unclear if these proteases are present in their active form in the respiratory system during infection, but they clearly have the ability to activate HA from human-adapted subtypes and, thus, may play a role in influenza infection in vivo.

The cleavage site sequence linking HA1 and HA2 is a crucial determinant for the pathogenicity and tropism of AIV in humans. Highly pathogenic avian influenza (HPAI) strains have a polybasic sequence at the HA cleavage site that allows for intracellular cleavage by ubiquitous subtilisin-like proteases such as furin [125]. As the host protease responsible for cleavage activation is widespread, the HPAI strains are not restricted to a particular tissue in this condition, which is thought to be one of the main reasons for the enhanced virulence. Conversely, low-pathogenic avian influenza (LPAI) strains feature a monobasic cleavage site that is cleaved by trypsin-like serine proteases that are either secreted into the extracellular space or present at the plasma membrane. As a result, the localisation of these proteases appears to limit the tissue tropism of LPAI. Although not well understood in avian species, equivalent host proteases for AIV in humans are thought to be primarily found in the respiratory system [126].

Moreover, N-glycosylation of the HA of AIV, which involves carbohydrate side chains linked to asparagine residues at the cleavage site of HA, is believed to significantly influence the proteolytic activation of HA [116]. The carbohydrate side chains on HA normally play a role in protecting the protein from being cleaved by enzymes such as trypsin [127]. However, in the absence of these side chains due to the mutation of asparagine at position 11, HA can be cleaved without the need for trypsin. This cleavage event can result in a shift to higher pathogenicity because it can allow the virus to infect host cells and spread more efficiently [128]. In contrast, in the HPAIV A/Mallard/Huadong/S/2005 (H5N1) virus, the removal of the same glycosylation site resulted in significantly delayed HA cleavability and lowered virus fitness in vivo and in vitro, while the experimentally modified glycosylation site was restored after a few viral replication cycles [129].

#### 5.2.2. HA and pH of the Host

Aside from host proteases and carbohydrate modification on HA, the membrane fusion activity of HA is influenced by the pH of the host cell. The association between HA and pH of the host cell is always attributed to HA activation pH and HA stability. The HA activation pH is the pH value at which membrane fusion occurs due to HA’s irreversible conformational change. In comparison, HA stability is defined as the ability of HA to resist inactivation after exposure to an extreme pH value [130]. All subtypes have an HA activation pH from 5.0 to 6.0, with avian viruses (H5N1, H7N7, H7N9, H9N2) having an average HA activation pH of 5.7 and human viruses (H1N1, H3N2) trending lower with an average of 5.4. Before infecting human airway epithelial cells, AIV virions must cross the respiratory mucosa, which has a pH as low as 5.5 [4,131]. At the low pH condition outside the targeted cell (as in the respiratory mucosa), the HA of AIV will be activated prematurely. This will result in the irreversible conformational change of HA, which will then cause the inactivation of the virions [130].

In order to avoid premature activation, the HA activation pH of AIV needs to be in the range between 5.0 and 5.5, which is slightly lower than the pH of respiratory mucosa, as this will increase HA stability for efficient transmission in humans. Avian H5N1 mutants with activated stabilized HA at pH 5.2 demonstrated efficient replication in the upper respiratory tract and airborne transmissibility in ferrets, similar to human influenza viruses [132]. The H17, Y17, H106_2_ and H111_2_ amino acid substitutions are the likely causes of the stabilized HA in H5N1 and other AIV subtypes [133]. It is generally recognized that electrostatic interactions between amino acids at interfaces, including hydrogen bonds, salt bridges and van der Waals interactions, have a significant role in HA’s stability, flexibility and functionality. For example, it was proposed that at a low pH, the protonated H106_2_ establishes a repulsive force with the K51_2_ at the stalk, which may be crucial for the folding of helix A following the development of the extended intermediate [133]. This repulsive force is lost when one of these amino acids is changed to a neutral amino acid, which lowers the fusion’s pH and stabilizes the HA.

### 5.3. Nuclear Import of vRNP

Upon membrane fusion, the newly released vRNP from the cytoplasm needs to be imported into the nucleus, since the replication and transcription of the viral genome by the viral RNA polymerase takes place inside there. In comparison to the earlier viral entry steps, the import of vRNP into the nucleus is highly dependent on the host cell machinery [134]. The nuclear import can be facilitated by factors, such as heat shock protein 90 (HSP90) [135], heat shock protein 40 (Hsp40/DnaJB1) [136], phospholipid scramblase 1 (PLSCR1) [137], translation elongation factor 1 delta (eEF1D) [138], nucleoporin 85 [139] and importin α [134], which tend to result in efficient and enhanced IAV replication. Among the host machinery involved in the nuclear import of vRNP, the interaction between importin α and viral NP is considered a key determinant of AIV tropism in humans [140,141].

#### NP and Host Importin α

Protein trafficking from the cytoplasm into the nucleus is a facilitated process driven by importin family members, which are divided into importin α and importin β based on their function [142]. Importin α recognizes the nuclear localisation signal (NLS) from molecules that leads to recruitment of importin β, which facilitates the transport of molecules into the nucleus through the nuclear pore complex (NPC) [134]. IAV hijacks this host machinery by equipping NP (which is a complex as vRNP) with an NLS that is recognized by importin α as an NLS-carrying cargo protein, and, thus, results in vRNP being transported into the nucleus by importin β [134].

Importin α is further categorized into a variety of isoforms, with the isoforms α1, α3, α4, α5, α6 and α7 being found in both avian and human hosts [140]. According to Gabriel et al., AIV prefers utilizing importin α3 (in avian host), whereas adapted human AIV prefers utilizing both importin α3 and importin α7 (in human host) to start the transport of viral proteins into the nucleus [140]. The fact that importin α7 is more widely distributed in the upper and lower respiratory tracts of humans than importin α3 suggests that the adapted human AIV may be under selective pressure to prefer importin α7 [7,143]. Hence, a retained preference to importin α3 and an additional preference towards importin α7 is needed by AIV to allow efficient nuclear import in human cells [140,143,144].

The adaptation of H7N7 NP towards importin α7 via a substitution from asparagine to lysine at location 319 (N319K) promotes viral growth and enhances pathogenicity in human cells [140]. This N319K substitution was also found in human isolates H5N1 [145], highlighting the possibility of this substitution in enhancing the nuclear import of vRNP and efficient replication of AIV in humans.

### 5.4. Replication and Transcription of Viral RNA

Inside the nucleus, the transcription and replication of viral RNA occur concurrently and are tightly linked processes that are dependent on each other. The viral RNA polymerase complex is responsible for both processes and is able to switch between transcription and replication modes as needed. Just after the inoculation in the nucleus, the viral RNA polymerase complex transcribes the viral RNA to produce mRNA, which is used to synthesise viral proteins. After the transport of these proteins into the nucleus, the replication process begins [134].

#### 5.4.1. PB2 and Host ANP32A

**Figure 6:**
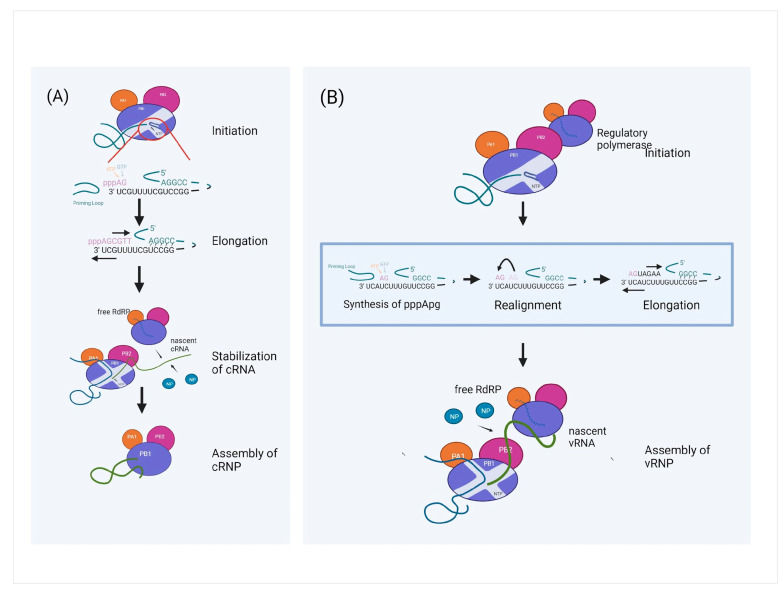
The replication of IAV viral RNA comprises two steps: (**A**) Replication of vRNA to cRNA. Initiation of replication occurs when PB1 of the RNA polymerase generates pppApg dinucleotide at position 1 and 2 of the 3′ UC region of the vRNA template. Priming loop is truncated and stimulates the elongation of the nascent cRNA by breaking the 5′ to 3′ base pair of the vRNA panhandle region. Next, the resulting cRNP complex is stabilized by new NP and free RdPR. (**B**) Replication of cRNA to vRNA. With the assistance of a free RdRP, replication of vRNA is activated with synthesis of pppApg at position 4 and 5 of the 3′ UC terminal of cRNA template. Dimerisation between cRNP RNA polymerase and a regulatory polymerase facilitate backtracking of the cRNA and cause realignment of the pppApg dinucleotide from position 4 and 5 to position 1 and 2. This initiates the elongation of the nascent vRNA. The nascent vRNA is stabilized by free RdRP and new NP. (Created with BioRender.com).

The replication of viral RNA takes place in the nucleus, where it comprises two-step processes as shown in Figure 6. In the first step, the viral RNA template is used to synthesise the complementary RNA intermediate (cRNA), while in the second step, the progeny vRNA is synthesized from the cRNA template [146]. The initiation starts when the RNA polymerase generates a pppApg dinucleotide at position 1 (3′ terminal UC of the vRNA template) which serves as a primer for replication, with the priming loop acting as a stabilizer for the initiation complex. Following initiation, the priming loop is truncated, stimulating the nascent cRNA’s elongation. In order to avoid cRNA degradation, the released nascent cRNA from the RNA polymerase binds to the new NP. It assembles with the terminal end of the cRNA and the newly synthesized RNA polymerase to form a complementary RNP (cRNP) complex. In contrast to cRNA synthesis, where pppApg is produced at position 1 of the vRNA template, pppApg during vRNA synthesis is produced at locations 4 and 5 of the cRNA template. The dimerisation between the cRNP RNA polymerase and a regulatory polymerase causes the priming loop to change position and facilitates the backtracking of the cRNA template. This contributes to the realignment of pppApg from positions 4 and 5 to positions 1 and 2 before acting as a primer to initiate vRNA synthesis [147]. As soon as the vRNA product leaves the active site of the cRNP-resident polymerase after elongation, the 5′ end is most likely grabbed by a stabilizing polymerase, starting the assembly of the vRNA with NP into vRNP [148].

Since AIV polymerases conduct genome replication poorly in humans [4], there are many mutations for the host adaptation, the best known of which is E627K in the PB2 that interacts with a host protein, acidic nuclear phosphoprotein 32 family member A (ANP32A) [149]. ANP32A belongs to a group of nuclear proteins involved in several cellular processes including messenger RNA export and the regulation of transcription [150]. It was proposed that the residue at location 627 of PB2 interacts with the host ANP32A to recruit a second polymerase for nascent vRNP formation [151]. The dependency of viral polymerase on ANP32A is essential to cross the host barrier from an avian to a human host [148]. Initially, avian ANP32A possesses a unique 33 amino acid insertion that is needed to support PB2 627E-containing polymerase. However, the lack of this insertion in human ANP32A restricts the AIV polymerase from replicating [152]. Interestingly, the E627K mutation in PB2 causes AIV polymerase activity to be supported by the shorter ANP32, resulting in AIV replication in humans [148]. All human circulating viruses of the 20th century carried the E627K mutation in the PB2, including the 1918 H1N1 pandemic virus, followed by the 1957 H2N2 and 1968 H3N2 pandemic viruses [7]. Alternatively, the 2009 pandemic virus retained E627 in the PB2 but with mutations of G590S and Q591R, which were close to position 627, explaining how it replicated efficiently in humans [4,153]. ANP32B, another ANP32 member, is also involved in IAV replication. Human ANP32B also supports AIV replication with the adaptation of E627K of the PB2 [154]. However, unlike human ANP32B, avian ANP32B is unable to support IAV replication due to amino acid mutations at positions 129 and 130 [155]. The E129A and D130A mutations in ANP32B were found to reduce the nuclear export of the influenza A virus vRNP complex, which is required for viral replication, and, thus, reduced the viral replication efficiency in mammalian cells [156]. This demonstrates the significance of ANP32B residues of 129 and 130 during IAV replication and suggests they are a potential target for the development of anti-influenza approaches.

#### 5.4.2. PA and Host RNA Polymerase II

Since influenza virus RNA polymerase cannot synthesise and methylate cap structures as non-segmented negative-sense RNA viruses do, viral RNA polymerases steal the cap-1 structure from the nascent host mRNA during viral transcription in the nucleus [148]. The transcription starts when the viral RNA polymerase interacts with the carboxy-terminal domain (CTD) of host RNA polymerase II (Pol II), which stabilizes the PB2 cap-binding and PA endonuclease domains before PB2 binds to the 5′ cap of nascent host mRNA and PA cleaves approximately 10–14 bases downstream from the cap [140]. Following that, the 3′ end of the cap base pairs with the 3′ end of the viral RNA template, which initiates elongation and duplex-winding until the viral RNA polymerase stops on a uridine sequence near the 5′ end of the viral RNA template. This terminates transcription by the addition of a poly(A) tail, which generates viral pre-mRNA [148].

The interaction with host factors during viral genome transcription highlights PA potential as a key determinant of AIV tropism in humans. The PA protein is structurally divided into the major domains of N-terminal (PA-N) and C-terminal (PA-C) [157]. The PA-N domain comprises the cap-snatching endonuclease and protease active sites, while the PA-C domain forms the core structure of the polymerase complex with PB1. The PA-C also has a site for 5′ vRNA binding and a site for host Pol II interaction, which together constitute the PA arch that functions to grab 5′-capped primers from nascent Pol II transcripts for transcription of viral mRNA. According to Arai et al., the PA-C localized mutations S388R and A448E contribute to the higher polymerase activity of H5N1 and expand the host range into humans [157]. PA residue 388 is a vital element of the 5′ vRNA binding site, located within the PA arch and the neighboring PB1 β-hairpin domain. Initially, it was found that S388 was involved in a hydrogen bond network with the PB1 protein; however, substituting R388 excluded the residue from the network. This suggests that the S388R substitution in PA creates flexibility at this location and there is a potential association with the vRNA promoter. Therefore, S388R may influence viral polymerase activity by modifying the interaction between the polymerase complex and the viral RNA promoter. Subsequently, PA residue 448 was found in helix α16 of the polymerase complex structure, which interacts with the PB1 domain, and this residue also directly interacts with the host CTD Pol II [158]. The A448E substitution forms a new hydrogen bond to N444, possibly stabilizing the helix α16 structure. Thus, it is suggested that the substitution of A448E may influence polymerase activity by altering the main structure of the polymerase complex or interacting with host Pol II. In the case of H7N9, the substitution of amino acid at location 409 from asparagine (N) to serine (S) increases the polymerase activity of the virus to replicate efficiently in humans and mammals [159]. The interaction between 409S and the PB1-N terminal region may be the cause of the increase in polymerase activity, and, as a result, this interaction may also enhance H7N9’s ability for human adaptation [159].

### 5.5. Maturation of Viral mRNA

After transcription, the viral mRNAs leave the nucleus to be transported to the host ribosome for translation. However, the viral pre-mRNA of M and NS goes through splicing (using host machinery), where introns (non-coding sequences) must be cut out to precisely link exons, the actual coding sequences, to generate mature mRNA before translation [41]. Influenza viruses hijack host splicing machinery to block host mRNA translation while utilizing the host spliceosome to allow the translation of specific spliced influenza virus mRNA.

#### M and Host huTRA2A

Many host-splicing regulators have been recognised as having a role in IAV mRNA splicing regulation, and one of them, host human transformer-2 protein homolog alpha (huTRA2A), has been reported as a determinant of AIV’s ability to adapt to humans [160]. huTRA2A is one of the host-splicing regulators, along with splicing factor 2 (SF2), heterogeneous nuclear ribonucleoprotein K (hnRNP K) and influenza virus NS1 binding protein (NS1-BP), that has been linked to the splicing of IAV mRNA [161]. SF2, hnRNP K and NS1-BP all promote virus replication in both humans and avians, but huTRA2A promotes virus replication in humans while inhibiting virus replication in avians [160].

As reported by Zhu et al., due to the RNA-recognition motifs of huTRA2A, it is able to bind the intronic splicing silencer (ISS) motif (GAAARGARR) of human IAV non-structural (NS) and AIV matrix (M) genes to stop mRNA splicing [160]. However, the binding generates two different outcomes. The inhibition of human NS mRNA splicing by huTRA2A produces a small but balanced ratio of NS1/NEP, which eventually increases viral polymerase activity and, thus, promotes human virus replication. On the other hand, the inhibition of AIV M mRNA splicing by huTRA2A leads to an imbalance in M2/M1 expression (less M2 and the same amount of M1), which in turn restricts AIV replication in humans, possibly because of the M2 role in virus budding. The results were confirmed by knocking down huTRA2A, which decreases human IAV replication while increasing AIV replication in human A549 cells. Human NS gene mutations 234G/236G and AIV M gene mutation 334M, both of which result in the loss of the ISS motif, have the same effect as the knockdown, decreasing human IAV replication while increasing AIV replication in human A549 cells. These findings indicate that huTRA2A is one of the determinants for AIV to cross the human barrier and that the AIV M gene substitution 334M is an important adaptive mutation that leads to a balance in M2/M1 expression for efficient replication in humans.

### 5.6. Nuclear Export of vRNP and Trafficking of Viral Proteins

The translation of IAV mRNA is carried out by cytosolic ribosomes (for PA, PB1, PB2, NP, NS1, NS2 and M1 transcripts) and endoplasmic reticulum-associated ribosomes (for HA, NA and M2 transcripts). All nascent proteins translated from the cytosolic ribosome will be imported back into the nucleus, while nascent HA, NA and M2 are trafficked through the Golgi to the plasma membrane. Once the copies of vRNA are generated in the nucleus, nascent NP binds to them, followed by the assembly of nascent PA, PB1 and PB2, forming vRNP [134]. Then, the vRNPs are exported from the nucleus with the assistance of M1, NP and NS2/NEP before being trafficked to the plasma membrane, where HA, NA and M2 are all located at the budding boundary.

#### 5.6.1. NP and Host CASP3

Caspase-3 (CASP3) is an enzyme involved in programmed cell death or apoptosis. A study has shown that CASP3 can be activated in response to IAV infection via the apoptotic pathways [162]. The activation of CASP3 can lead to the cleavage of viral proteins, including NP.

The NP of IAV can be cleaved by CASP3 at a specific location, METD/16, which is highly conserved among human IAVs [163]. This NP cleavage produces a small N-terminal fragment of 3 kDa (residues 3 to 13) that contains a nuclear localisation signal (NLS) [164]. The removal of this NLS by CASP3 likely allows the assembled ribonucleoprotein (RNP) complex to be transported to the cytoplasm, which is necessary for viral replication [165]. Inhibition of CASP3 results in decreased viral replication due to the retention of vRNP in the nucleus [162]. Interestingly, AIV has a different NP sequence that lacks the caspase’s cleavage motif and, therefore, it is uncleavable by human CASP3 [165]. Thus, we anticipate that this difference might be a potentially key point in determining the host tropism of AIV in humans.

#### 5.6.2. RdRp Complex and Host SERTAD3

SERTAD3 is a transcription factor belonging to the SERTA family. In normal cells, SERTAD3 knockdown significantly reduces a cell’s growth rate, whereas SERTAD3 overexpression causes oncogenic transformation, implying a role in cellular growth [166]. Regarding this protein’s role in the infection and immune response, SERTAD3 interacts with the African swine fever virus protein MGF360-16R [167] and is involved in the late-phase response of toll-like receptor (TLR) signaling [168].

According to transcriptomic data from infected A549 cells, SERTAD3 was the most significantly expressed gene among the four SERTAD family members in response to H5N1 infection [169], indicating that SERTAD3 may function as an interferon-stimulated gene (ISG). Similar to many other ISGs, Sun et al. postulated that SERTAD3 might be involved in antiviral signaling by participating in retinoic acid-inducible gene I (RIG-I)-like receptor (RLR) signaling [169]. Not long after, they discovered that SERTAD3, rather than playing a role in RLR signaling, suppresses AIV replication by interacting with the PB1, PB2 and PA, and blocking the assembly of the functional RdRp complex. To demonstrate that SERTAD3 does not merely bind any overexpressed protein, NA and NP were utilized as negative controls, and no interaction was observed between SERTAD3 and either NA or NP. Additionally, immunofluorescence staining of SERTAD3 with PB1, PB2 or PA following virus infection revealed that all three of these proteins co-localized with SERTAD3 in the nucleoli [169].

In addition, a peptide containing eight amino acids (labelled as AD-C) found in the C-terminal activation domain of SERTAD3 is important in limiting AIV replication [169]. The deletion mutants missing the C-terminal activation domain lost the inhibitory activity of SERTAD3 (increased AIV replication), and overexpression of the AD-C reduced H5N1 minireplicon activity. We speculate that if AIV acquires an adaptive mutation that neutralizes this 8-amino acid peptide, SERTAD3’s inhibitory function during the assembly of the RdRP complex will be blocked, allowing AIV to replicate in humans efficiently.

### 5.7. Release

Once all the new viral components are situated at the budding boundary within the membrane, IAV alters the structure of the membrane to initiate bud formation and, finally, the scission of the new virion envelope from the membrane occurs, allowing it to infect other host cells [134]. Multiple studies have suggested that the involvement of M1 increases the budding efficiency and shape uniformity of the newly produced virion envelope, and that the expression of HA and NA is sufficient to induce budding and facilitate the virus’s release [170,171,172]. The adaptation of three viral proteins, HA, NA and M1, which interact with the host factors, including the G protein subunit beta 1 (GNB1) and sialic acid receptor, promotes the efficient release of AIV from infected human cells.

#### 5.7.1. M1 and Host GNB1

M1 appears to be involved in the viral membrane-bending process during the release step. Its recruitment to the membrane budding site, where it oligomerizes upon reaching the membrane, forms a curvature structure and significantly promotes bud formation [134]. The recruitment of M1 to the membrane budding site is believed to be mediated by GNB1, a G protein subunit that mediates transmembrane signaling pathways that initiate membrane ruffling-like processes that result in virus budding [173].

As reported by Li et al., there is likely no interaction between host human GNB1 and AIV M1, indicating that M1 is not being recruited by GNB1 to the budding site [173]. However, they discovered that human GNB1, specifically in conjunction with the AIV reassorted mutant (H5N6 with H9N2 virus-derived M1), stimulated virus budding, as GNB1 binds to M1 to form a complex before co-transporting it to the viral assembly and budding site. Hence, the reassortment of genes involving AIV M1 in association with the host GNB1 successfully initiates efficient budding and release in human cells.

#### 5.7.2. NA and Host SA

As soon as the recently assembled virion buds, its release is heavily reliant on the enzymatic activity of NA [134]. NA is a homotetramer, and its homotetramer structure is important for forming a deep pocket containing the active site at the globulin head [174]. NA promotes viral release by cleaving the glycosidic linkage that connects sialic acid (SA) to the penultimate galactose via the action of residues at the active site [134]. By eliminating the SA, NA blocks HA from binding to the surface membrane, thereby enabling effective virus release during budding.

AIV release in humans appears to be inefficient due to the aggregation of the new virion at the surface membrane, which is most likely caused by the very low enzymatic activity of NA [175]. The low enzymatic activity of NA is most probably because of the lack of specificity between the shape of the active site and the substrate [176]. Like HA, AIV NA’s active site is specific to α2,3 SA, and this specificity excludes human SA with the α2,6-linkage [107]. To properly release AIV from the human surface membrane, the virus must modify its NA active site to become specific to the SA via α2,6-linkage, hence, enhancing NA enzymatic activity. A study comparing fatal and conjunctivitis cases of H7N7 infection in humans discovered that four residue substitutions in NA, N308S, A346V, T442A and P458S, increase the NA enzymatic activity, resulting in efficient virus release and increasing virus titers [177]. Except for residue 458, which is located at the C-terminus of NA [178], all of the substituted residues are located near the active site of NA [179,180,181] and may affect the enzymatic activity.

Another aspect that contributes to the efficient release of AIV from the human surface membrane is the balance of HA–NA activity [92]. As HA of AIV has a poor binding to the α2,6 SA, a substitution in the second SA binding site in the NA of the new virion enables the enzymatic activity of NA to be compatible with the poor HA–SA binding, thus, leading to efficient release and replication in humans [174].

### 5.8. Evasion of the Host Immune Response

AIVs have developed multiple strategies to evade the cellular innate immune response, enabling the viruses to replicate and complete their life cycle. One of the key strategies employed by AIV is the production of NS1 protein, a multi-functional non-structural protein encoded on segment 8 of the IAV viral RNA. NS1 is an important virulence factor that plays a crucial role in the virus life cycle by thwarting the host immune response. NS1 counteracts the host’s antiviral defenses through the production of interferon (IFN) and antiviral IFN-induced proteins, such as dsRNA-dependent protein kinase R (PKR) and RNase L (IFN). The AIV’s NS1 protein deploys two methods to limit interferon production in human cells. The first is counteracting the activation of retinoic acid-inducible gene I RIG-I receptor through the ubiquitin ligase tripartite motif-containing protein 25 (TRIM25), while the second is binding to CPSF30, a protein that processes newly synthesized mRNAs, including IFN-β mRNAs [4,182]. Despite NS1’s ability to bind to various host factors, mutations in NS1 are not typically linked to the host’s range. In fact, most AIV NS1 proteins can effectively inhibit interferon production in human cells by binding to human CPSF30. In mice, a P42S mutation in the NS1 protein of the H5N1 strain significantly increased its virulence. This mutation was also present in the H7N9 virus that was circulating. When combined with other mutations in HA, NA, PB1 and PB2, it contributed to the pathogenicity of the AIV H7N9 strain in humans [183].

Recent studies have suggested that NS1 may also play a role in determining the host tropism of avian influenza viruses. For instance, Evseev et al. conducted a study that demonstrated NS1 proteins from both low-pathogenic and highly pathogenic avian influenza viruses can effectively inhibit RIG-I ubiquitination and reduce interferon promoter activity and interferon-beta protein secretion in transfected human cells, while the NS1 of the mouse-adapted PR8 strain does not have this ability. However, when the NS1 proteins were cloned into recombinant viruses and used to infect alveolar cells, all NS1 proteins successfully suppressed interferon in the infected cells. Interestingly, the avian NS1 proteins did not suppress duck RIG-I ubiquitination and interferon promoter activity, despite interacting with duck TRIM25 [183].

## 6. Conclusions

Many subtypes of AIV have been confirmed to infect humans including H3N8, H5N1, H5N6, H5N8, H6N1, H7N1, H7N2, H7N3, H7N4, H7N7, H7N9, H9N2, H10N3, H10N7 and H10N8 [184,185]. Among these were the highly virulent subtypes H5N1 and H7N9, as well as H5N6, which caused high mortality in humans. Since its first appearance, H5N1 has been linked to more than 800 cases and 400 deaths globally, H7N9 has been responsible for more than 1500 human cases and 600 deaths worldwide, while 48% of H5N6 cases are fatal in humans [71,186]. Most AIV subtype infections were caused mainly through exposure to infected poultry, suggesting that human-to-human transmission of AIV is still ineffective. However, given what we currently know about the rapid adaptation of AIV, efficient human-to-human transmission is possible, and it is just a matter of time before it happens.

Therefore, we have highlighted the key determinants for AIV tropism in humans according to the most frequently reported virus and host factors. Based on that, we suggest the minimal requirement for efficient AIV transmission between humans that could initiate new pandemics: (i) an adaptation of the receptor binding preference of HA to α2,6 SA during attachment; (ii) optimum HA stability during membrane fusion; (iii) the improvement of efficiency during the import of vRNP into the nucleus; (iv) the increase of polymerase activity and flexibility during transcription and replication; (v) the balance in M2/M1 expression after M segment splicing; (vi) efficient assembly of the RdRP complex; (vii) cotransport of the M1–GNB1 complex to the budding site; (viii) a functional balance between HA affinity and NA enzymatic activity with SA; (ix) efficient NS1-mediated evasion of the host immune response.

It is necessary to be prepared for the worst, since we cannot predict what types of mutations may occur in the future that could potentially cause pandemics. Understanding the mechanisms that underlie adaptive changes and the circumstances in which they can be acquired will enhance our ability to assess the public health risks posed by AIV and predict the source of future pandemics. It will also reveal intricate details of virus–host interactions that can lead to the development of novel vaccines, antiviral agents and effective therapeutic strategies.

## Figures and Tables

**Table 1:** Influenza A virus gene segments and proteins’ size and functions. ^1^ RNA-dependent RNA polymerase (RDRP); ^2^ Auxiliary proteins.

Segment	Segment Size (Nucleotides)	Proteins	Protein Sequence (Amino Acids)	Viral Function	References
1	2341	PB2	759	RDRP ^1^ subunit: transcription initiation and cap-snatching mechanism	[31,32]
2	2341	PB1	757	RDRP ^1^ subunit: replication and transcription of viral RNA segments; endonuclease activity	[26,33]
		PB1-F2 ^2^	87–90	Proapoptotic activity contributes to the pathogenicity of IAVs	[31,34]
		PB1-N40 ^2^	718	Regulation of PB1 expression and activity	[28,33]
3	2233	PA	716	RDRP ^1^ subunit: endonuclease, cleaves the capped RNA	[31]
		PA-X ^2^	252	Role in influenza virus-induced host shutoff as it selectively degrades host RNAs and limits innate immune responses	[35,36]
		PA-N155 ^2^	562	Role in viral replication	[37,38]
		PA-N182 ^2^	535	Role in viral replication	[37,38]
4	1778	HA	566	Binding to cell receptor; fusion of endosomal and viral membranes	[39]
5	1565	NP	498	Transcription/replication regulation, vRNP nuclear export	[26,39]
6	1413	NA	454	Sialidase activity (propagation of neovirions)	[39,40]
7	1027	M1	252	Support of structure and internal viral architecture; regulation of RNA nuclear export activity	[26,31,37]
		M2	97	Ion channel activity, as well as a role in virus uncoating and assembly	[26,31,37]
		M42 ^2^	99	A functional alternative to M2	[37,41]
8	890	NS1	215–237	Inhibition of the antiviral response	[39,42]
		NS3 ^2^	194	Unknown function	[31]
		NS2/NEP	121	Nuclear export of new vRNP	[43]

**Table 2:** Summary of all human infections caused by the avian influenza viruses.

AIV Subtype	Year	Location	Cases/Fatalities	First Isolated Human Strain	References
H3N8	2022	China	2/0	A/Henan/4-10/2022 A/Changsha/1000/2022	[66]
H5N1	1997	Hong Kong	18/6	A/Hong Kong/156/97	[68]
	2003	Hong Kong	2/1		[69]
	2003	China	1/1		[70]
	2003–January 2023	21 countries	868/457		[71]
H5N6	2014–February 2023	Western Pacific Region	83/33	A/Sichuan/26221/2014	[71]
A(H5) *	October 2022	Vietnam	1/1		[71]
H5N8	2020	Russia	7/0	A/Astrakhan/3212/2020	[72]/[73]
H6N1	2013	Taiwan	1/0	A/Taiwan/2/2013	[74]
H7N2	2002	USA	1/0	A/New York/107/2003	[75]
	2003	USA	1/0		[75]
	2007	UK	4/0		[76]
	2016	USA	1/0		[77]
H7N3	2004	Canada	2/0	A/Canada/444/04	[78]
	2006	UK	1/0		[79]
	2012	Mexico	2/0		[80]
H7N4	2018	China	1/0	A/Jiangsu/1/2018	[81]
H7N7	1996	UK	1/0	A/England/268/96	[82]
	2003	Netherlands	89/1		[83]
	2013	Italy	3/0		[84]
H7N9	2013–February 2023	Western Pacific Region	1568/616	A/Anhui/1/2013	[71]
H9N2	1998	China	5/0	A/HK/1073/99	[85]
	1999–2009	Hong Kong	6/0		[64]
	2011	Bangladesh	1/0		[86]
	December 2015–February 2023	Western Pacific Region	82/2		[69]
H10N3	2021	China	2/0	A/Jiangsu/428/2021	[87]
H10N7	2004	Egypt	2/0	A/Egypt/2004	[88]
	2010	Australia	2/0		[89]
H10N8	2013	China	3/2	A/Jiangxi-Donghu/346/13	[90]

* The NA subtype could not be determined.

## Data Availability

Data sharing not applicable.
